# Using Deep Learning and Resting-State fMRI to Classify Chronic Pain Conditions

**DOI:** 10.3389/fnins.2019.01313

**Published:** 2019-12-17

**Authors:** Alex Novaes Santana, Ignacio Cifre, Charles Novaes de Santana, Pedro Montoya

**Affiliations:** ^1^Research Institute of Health Sciences (IUNICS-IdISBa), University of the Balearic Islands, Palma, Spain; ^2^Facultat de Psicologia, Ciències de l’Educació i de l’Esport, Blanquerna, Universitat Ramon Llull, Barcelona, Spain; ^3^Landscape Ecology, Institute of Terrestrial Ecosystems, ETH Zürich, Zurich, Switzerland

**Keywords:** chronic pain, machine learning, classification, rs-fMRI, deep-learning, DTW

## Abstract

Chronic pain is known as a complex disease due to its comorbidities with other symptoms and the lack of effective treatments. As a consequence, chronic pain seems to be under-diagnosed in more than 75% of patients. At the same time, the advance in brain imaging, the popularization of machine learning techniques and the development of new diagnostic tools based on these technologies have shown that these tools could be an option in supporting decision-making of healthcare professionals. In this study, we computed functional brain connectivity using resting-state fMRI data from one hundred and fifty participants to assess the performance of different machine learning models, including deep learning (DL) neural networks in classifying chronic pain patients and pain-free controls. The best result was obtained by training a convolutional neural network fed with data preprocessed using the MSDL probabilistic atlas and using the dynamic time warping (DTW) as connectivity measure. DL models had a better performance compared to other less costly models such as support vector machine (SVM) and RFC, with balanced accuracy ranged from 69 to 86%, while the area under the curve (ROC) ranged from 0.84 to 0.93. Also, DTW overperformed correlation as connectivity measure. These findings support the notion that resting-state fMRI data could be used as a potential biomarker of chronic pain conditions.

## Introduction

Pain is a subjective psychological phenomenon that emerges from brain activity but can be influenced by several and different aspects of human behavior and cognition (attention, learning, beliefs, etc.) (Albe-Fessar et al., 1985; Montoya et al., 2004). Indeed, pain is a complex problem in which biological, psychological, and social factors may play a relevant role in its maintenance over time (Bevers et al., 2016). This multidimensional aspect of pain requires that measurement of pain should include not only subjective ratings, but also psychological and neurophysiological events related to pain perception. Accordingly, research on cognitive pain has been conducted using several approaches and tools such as questionnaires (Pagé et al., 2015; Traeger et al., 2016), Quantitative Sensory Tests (QST) (Cruz-Almeida and Fillingim, 2014; Cámara et al., 2016), genetic factors (Diatchenko et al., 2005; Ablin and Buskila, 2015; Ultsch et al., 2016), patterns of physical activity (Hidalgo et al., 2012, 2014; Paraschiv-Ionescu et al., 2012), EEG (Pinheiro et al., 2016), neuroimaging (Davis et al., 2012; Schmidt-Wilcke, 2015), and more recently functional near-infrared spectroscopy (fNIRS) (Lopez-Martinez et al., 2018, 2019).

Chronic pain is characterized by symptoms such as pain that lasts more than 3–6 months (Merskey et al., 1994; Wolfe et al., 2016), as well as by fatigue, sleep disturbance, cognitive and mood changes (Gatchel et al., 2010). As a result, chronic pain may limit individual daily activities, leading to disability and reduced quality of life. Several studies have shown that chronic pain syndromes are also associated with alterations in the functional connectivity of BOLD signals (Baliki et al., 2008). For instance, chronic back pain (CBP) patients seem to have reduced deactivations in specific brain regions of the default mode network (DMN), such as mPFC, amygdala, and PCC. Furthermore, studies in patients with neuropathic pain (NP) have confirmed reduced DMN connectivity together with enhanced resting-state functional connectivity among several pain related areas (Cauda et al., 2009). These findings are in line with previous data suggesting that these alterations could be the neurophysiological mechanisms involved in cognitive and behavioral impairments associated with chronic pain (Apkarian et al., 2004; Cauda et al., 2009). The analyses of resting-state fMRI have suggested that the brain of chronic pain patients differ from that of healthy subjects by showing significant reductions of functional connectivity of the DMN, together with significant enhancements of several pain-related areas. Pain-related brain structures also presented significant changes in functional connectivity when comparing fibromyalgia (FM) patients and healthy controls (Cifre et al., 2012). FM patients show changes in regions involved in facilitating and reducing pain processing. Other similar studies using fMRI also found alterations in brain connectivity for patients with chronic prostatitis/chronic pelvic pain syndrome (CP/CPPS) (Johnson et al., 2015; Lin et al., 2017), migraine (Lovati et al., 2016), and other (Davis and Moayedi, 2013; Napadow and Harris, 2014; Truini et al., 2016; Zamorano et al., 2017). The intensity of these brain alterations is also correlated with pain intensity reported by CP patients (O’Shea et al., 2015). Also, patterns of functional brain connectivity have been widely investigated as a potential biomarker for classification and prediction of a variety of other neurological and psychiatric syndromes such as attention deficit hyperactivity disorder (ADHD), schizophrenia, and Mild Cognitive Impairment (Du et al., 2018).

Despite recent findings about neurophysiological mechanisms (central sensitization, brain plasticity), chronic pain remains under-diagnosed and under-treated. In some cases, more than 75% of the patients do not receive an accurate diagnosis (Kress et al., 2015; Dodick et al., 2016). Furthermore, the assessment of knowledge, attitudes, and beliefs (KAB) about chronic pain among primary care providers revealed that even those who participate in continuous education programs still may have inappropriate beliefs and attitudes about pain and its treatment (Lalonde et al., 2014). Therefore, one possible solution would be to provide clinicians with objective methods to support their decision about pain diagnosis and treatment. In this regard, a multidimensional framework and operational diagnostic criteria for the major chronic pain conditions were proposed by the Analgesic, Anesthetic, and Addiction Clinical Trial Translations, Innovations, Opportunities, and Networks (ACTTION), the US Food and Drug Administration, and the American Pain Society (APS) (Dworkin et al., 2016). This framework is divided into five dimensions: (1) Core Diagnostic Criteria, (2) Common Features, (3) Common Medical and Psychiatric Comorbidities, (4) Neurobiological, Psychosocial, and Functional Consequences, and finally (5) Putative Mechanisms, Risk Factors, and Protective Factors. Following this framework, other studies proposed evidence-based diagnostic criteria for specific chronic pain conditions (Dampier et al., 2017; Paice et al., 2017; Widerström-Noga et al., 2017; Zhou et al., 2018; Arnold et al., 2019; Freeman et al., 2019; Ohrbach and Dworkin, 2019). The majority of these criteria are composed by the patient historical data, self-reported information via questionnaires, and psychophysical tests that determine pain features and pain thresholds. Although pain neuroimaging was not presented in these criteria, it has been used in clinical trials with a focus on diagnostic properties of different conditions, including FM and chronic back pain (Smith et al., 2017).

Due to the complexity and the great number of features, the patterns of functional brain connectivity patterns are commonly analyzed using multivariate analysis such as Support Vector Machines (SVM), Logistic Regression (LR), and Least Absolute Shrinkage and Selection Operator (LASSO). In fact, the majority of the models mentioned in Du et al. (2018) made use of SVM. Deep learning (DL) comprehends a family of machine learning algorithms that use a set of processing layers to extract and transform features from data with multiple levels of abstraction. These algorithms have improved the image and speech recognition, compared with traditional machine learning algorithms (e.g., SVM and LASSO) (Krizhevsky et al., 2012; LeCun et al., 2015). This performance puts DL models in focus as a promising approach to classify brain images, especially for single subject prediction (Vieira et al., 2017). Furthermore, some DL architectures have been specially designed to learn from brain functional connectivity networks (Kawahara et al., 2017; Meszlényi et al., 2017). In the context of chronic pain, functional brain images were also used as an information source to multivariate pattern analysis in the attempt to classify or predict chronic pain syndromes (Callan et al., 2014; Sundermann et al., 2014; López-Solà et al., 2017). In these works, only traditional models such as SVM, LR, and k-Nearest Neighbors (k-NN) were trained to differentiate healthy control subjects from patients with FM (Sundermann et al., 2014; López-Solà et al., 2017) and from chronic back pain (CBP) (Callan et al., 2014). This restraint to traditional algorithms motivated us to apply DL to chronic pain classification. Moreover, the majority of such previous studies used a certain type of stimulus during the acquiring process, which requires a more adequate environment and a team of technicians to be executed. When a resting-state protocol was applied, the results had a lower performance with accuracy indices below 80%. Also, most of the studies focused on identifying only one chronic pain syndrome (usually CBP or FM).

Our objective in this work was to evaluate the performance of a set of DL algorithms in the classification problem of chronic pain syndromes and to compare it with the performance of traditional classifiers. Moreover, we will analyze how different brain parcelations and connectivity measures affect the classification performance. In order to achieve that, we analyzed the data using four different parcelations including ROI and group-ICA based parcelations, and two different measures of functional brain connectivity, such as correlations and Dynamic Time Warping.

## Materials and Methods

### Participants

The participants of the study were ninety-eight healthy controls (age: 40.85 ± 23.7) and sixty chronic pain patients (age: 45.65 ± 15.23). Sixty-four females (age: 39.33 ± 20.5) and thirty-four males (age: 43.76 ± 23.89) participants compose the healthy control group. A limitation of this study is that those male participants were presented only on the control group. The chronic pain group (CP) was composed of thirty-six FM patients and twenty-four chronic back pain patients. All chronic pain participants suffered from persistent pain for more than 6 months, also they were diagnosed following the IASP’s criteria (Merskey et al., 1994) and (Wolfe et al., 2016) for FM. There was no significant difference in age between the groups (*T* = 1.56, *p* = 0.11). No participant used opiates, gabapentin, or pregabalin for pain treatment. Four patients occasionally used non-steroidal anti-inflammatory drugs (NSAIDs) and/or paracetamol. Medication for non-pain related disorders involved in birth control and female hormonal drugs. Three CP took benzodiazepine (1–5 mg per day), of which one also took serotonin reuptake inhibitors.

### Image Acquisition

BOLD resting-state functional magnetic resonance images (rsfMRI) from one hundred and fifty-three subjects were used in this work. All data were collected using a GE 3T scanner (General Electric Signa HDx, GE Healthcare, Milwaukee, WI, United States). A total of 240 whole-brain echo-planar images were acquired in a time of 10 min with the following scanner parameters: 32 slices per volume, 3 mm of slice thickness, 4 mm of space between slices, repetition time equals 2500 ms, echo time equals 35 ms, 90° flip angle, matrix dimensions equal 64 × 64, and FOV equals 240. The structural images were collected using three similar protocols. These protocols differed exclusively by the number of slices per volume; where the values of 292, 220, 256, and 248 slices were used for 24, 44, 48, and 37 of all dataset, respectively. The other parameters were configured as follows: 1 mm of slice thickness, repetition time equals 7796 ms, echo time equals 2984 ms, 12° flip angle, matrix dimensions equal 256 × 256, and FOV equals 256. Scanner noise was passively reduced by −36 dB using in-ear hearing protection. In addition, MRI foam-cushions were placed over the ears to restrict head motion and further to reduce the impact of scanner noise.

### Image Preprocessing

Image preprocessing was performed using the Neuroimaging in Python Pipelines and Interfaces (Nipype) (Gorgolewski et al., 2011). The first five image volumes were excluded prior to image preprocessing. Then, spikes were removed using an algorithm from the Analysis of Functional NeuroImages (AFNI)^1^ software suite. Subsequently, the processes of slice-time correction, realigning, and coregistration were performed using the Statistical Parametric Mapping software package version 12 (SPM12). Also, the time-course signal-to-noise ratio (TSNR) of each time series was calculated together with a Nipype’s algorithm to detect artifacts based on RapidArt^2^. This algorithm uses intensity and motion parameters to infer outliers. Finally, all images were normalized to the standard Montreal Neurological Institute (MNI) stereotactic space with a bias regularization of 10^–4^ and a FWHM of Gaussian smoothness of bias of 60, resulting in a voxel size of 2 mm.

### Brain Functional Parcelation

We tested four different parcelation maps in order to identify how the number of parcels affects the classification process. Two parcelations were part of the UK Biobank Imaging Study^3^, where about five thousands resting-state functional MRI data points were collected from different participants. Both parcelations were the result of a group independent components analysis (group-ICA) (Miller et al., 2016). These parcelations were composed of 25 and 100 components. After the authors excluded the components that are not neuronally driven, the parcelations presented 21 and 55 components, respectively. Only this final sets of 21 and 55 components were used in our study.

**FIGURE 1:**
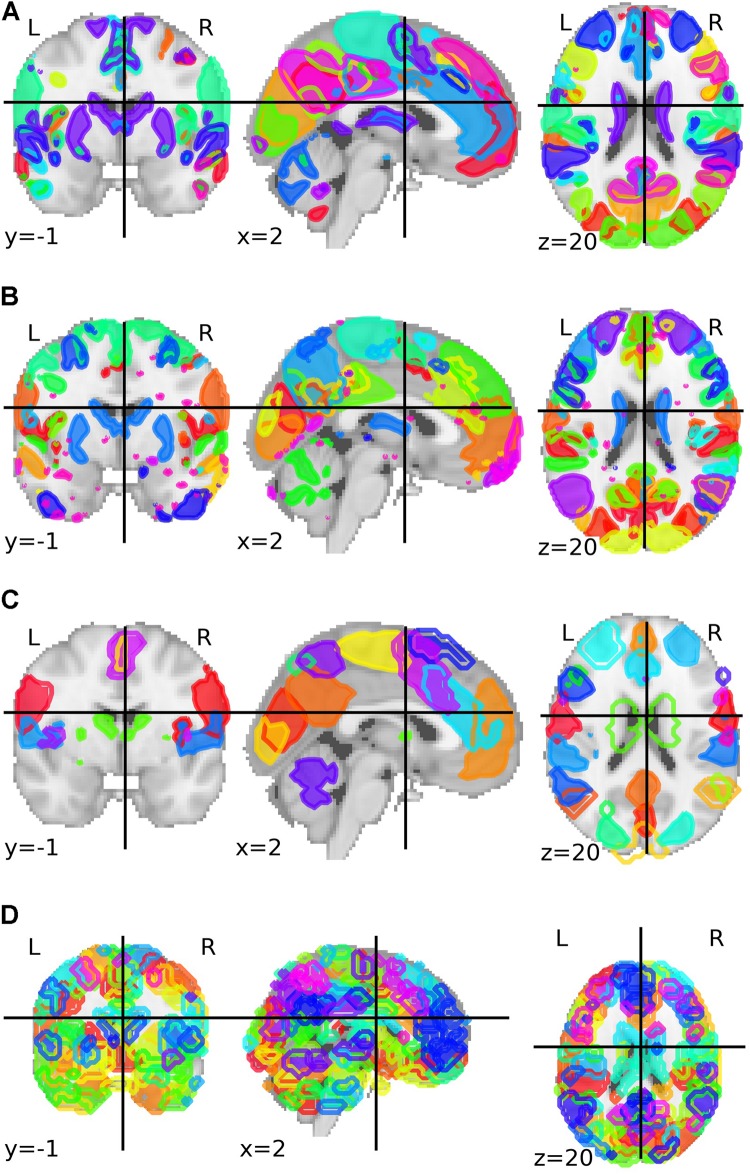
Group-ICA based parcelation from Biobank UK with **(A)** 21 and **(B)** 55 components after the authors excluded the components that are not neuronally driven. **(C)** Parcelation with 39 parcels and based on a multi-subject dictionary learning process (MSDL). **(D)** Willard functional atlas with 499 regions of interest.

The third parcelation was obtained from an analysis called Multi-Subject Dictionary Learning (MSDL) using resting-state functional MRI from 20 subjects with eyes closed (Varoquaux et al., 2011). The final map presented a parcelation with 39 components. The fourth and last parcelation was the Willard functional atlas, containing 499 functional regions of interest (Richiardi et al., 2015). All brain parcelations can be visualized in Figure 1.

### Extracting Connectivity Measures

The brain functional network of each participant was determined using the suite Nilearn (Abraham et al., 2014). This process followed a pipeline of three tasks. The first task was to clean and to exclude bad images: images that presented more than 3 “bad” slices detected by the artifacts detection algorithm were removed from the dataset. For the images remained, the time-series were detrended; the movement confounds were removed based on a projection on the orthogonal of the signal space (Friston et al., 1994; Lindquist et al., 2019); then, the images were standardized. The second task was to extract the brain activity time-series from the brain regions defined by the parcelation map. For each region, the least-squares solution was calculated, and a new time-series representing the region was obtained.

In the last task, a functional network was constructed using the brain parcels as nodes. The connectivity between each parcel was calculated using the representative time-series of each parcel, pair-wisely. Correlation and dynamic time warping distance (DTW) were used to measure the connectivity value between these time-series. DTW is a similarity measure for time-series, where one time-series can be “compressed” or “stretched” in time in order to find the best alignment with another time-series. It was first designed for speech recognition (Sakoe and Chiba, 1978) and was significantly more accurate than other similarity measures (Wang et al., 2013). In our experiments, the DTW was calculated using the FastDTW algorithm (Salvador and Chan, 2007). Finally, after calculating the connectivity matrix, we normalized it. We calculated the z-score of each matrix cell in regards with all cells. At this point, each image was converted into eight matrices, one for each combination of four parcelations (Biobank_UK_25, Biobank_UK_100, MSDL, and Willard) with two measures (Correlation and DTW).

### Classifiers

Four classifiers were compared to determine the one that presented the best results in a binary classification problem: chronic pain patients against controls. Three of the four classifiers used convolution neural network approaches. The classifier BrainNetCNN was defined by Kawahara and collaborators (Kawahara et al., 2017) and it proposes three new conventional filters. Those filters are adapted to adjacent matrices that represent any kind of neural network. The edge-to-edge filter computes a weighted response over neighboring edges for a given edge, while the edge-to-node filter computes a weighted response over neighboring edges for a given node. The third filter, called node-to-graph, applies a one-dimensional convolution to calculate a scalar based on a weighted combination of the nodes. Also, this architecture includes three fully connected hidden layers, characterizing it as a deep neural network.

The second classifier was created by modifying the BrainNetCNN. Batch Normalization layers were inserted between the BrainNetCNN layers to create a new classifier, called BrainNetCNNBatch. This classifier normalizes the activations of the previous layer at each batch, improving the performance and stability, and allowing to reach higher learning ratios (LRs) (Ioffe and Szegedy, 2015). In a similar work, Meszlényi et al. (2017) presented a convolutional neural network architecture to classify rsfMRI. The presented architecture was our third classifier, which had a sequence of two one-dimensional convolutional layers followed by a fully connected layer and a softmax layer with two outputs. See Supplementary Figure 1 for a detailed scheme of these convolution neural networks.

The fourth classifier was an automated machine learning toolkit called TPOT^4^. This classifier uses genetic programming to find an optimized machine-learning pipeline (Olson et al., 2016a, b). This toolkit allowed us to test different classical machine learning models and feature engineering processes with a reduced computational cost.

### Training and Evaluation

Before the training, the missing data were dropped, which together with the previous exclusion criteria of a maximum of 3 “bad” slices per image resulted in a dataset of 140 participants. Next, the dataset was split into training and testing dataset, keeping the original proportion of healthy controls and chronic pain patients. Training and testing datasets had 98 and 42 participants, respectively. Due to this reduced number of participants, we opted to augment the training data by creating five thousand Local Synthetic Instances (LSI) (Brown et al., 2015). This split 98/42 is the independent fold selection. These 42 subjects were separated in the beginning, before any data training or feature engineering. On the other hand, the 98 subjects from training dataset were split in the same proportion, if the model is a CCNN we did a single split (train/validation), otherwise (TPOT case) we applied a k-fold cross-validation approach. All scores presented in this paper are related to the independent fold. For CCNN, we opted to use only one split (train/validation/test) because of the computation cost. Also, during the data augmentation, the training and validation data were augmented separately to avoid any leak of information between the two sets.

Overfitting is a common problem during the development of a new machine learning model. It directly affects the model’s generalization, in other words, the ability to predict new scenarios (inputs) correctly. Overfitting can be detected by evaluating the model error during training and test. When the difference between training and test errors increase, it is a sign that the model is starting to overfit the training dataset. One approach to avoid this scenario is to use regularizers. Regularization is defined as any modification to a learning algorithm that is intended to reduce its test error but not its training error (Goodfellow et al., 2016, p. 177). Three kinds of regularizers are commonly used in neural network-based classifiers: Dropout, L1, and L2 regularizers.

In this work, different combinations of Dropout, L1, and L2 regularizers were tested. Dropout had values of [0, 0.3, 0.5, 0.8], these values represent the probability of a node be excluded from the neural network. This behavior generates sub-networks that are trained which permits we apply inexpensive bagged ensemble methods with neural networks. L1 and L2 are also called Lasso regression and Ridge regression, respectively. These two regularizers add a penalization term to the model’s loss function. To control how this term affects the loss function, a constant λ is multiplied to it. For λ we had values of [0, 0.1, and 0.01]. When gamma assumes a value of zero, this regularizer is deactivated. The range values of dropout and λ for L1 and L2 were chosen arbitrarily. Also, when not specified in the original architecture description, the regularization was applied between the fully connected layers.

The LR was another parameter that was optimized and tested with values of [0.1, 0.01, and 0.001]. Thus, each combination of parcelation, connectivity measure (Correlation and DTW), neural network architecture, learning ration, and regularizers created a classification experiment, combining into a total of 2592 classification experiments. These experiments were trained using the five thousand synthetic instances and evaluated using forty-two participants from the testing set.

**FIGURE 2:**
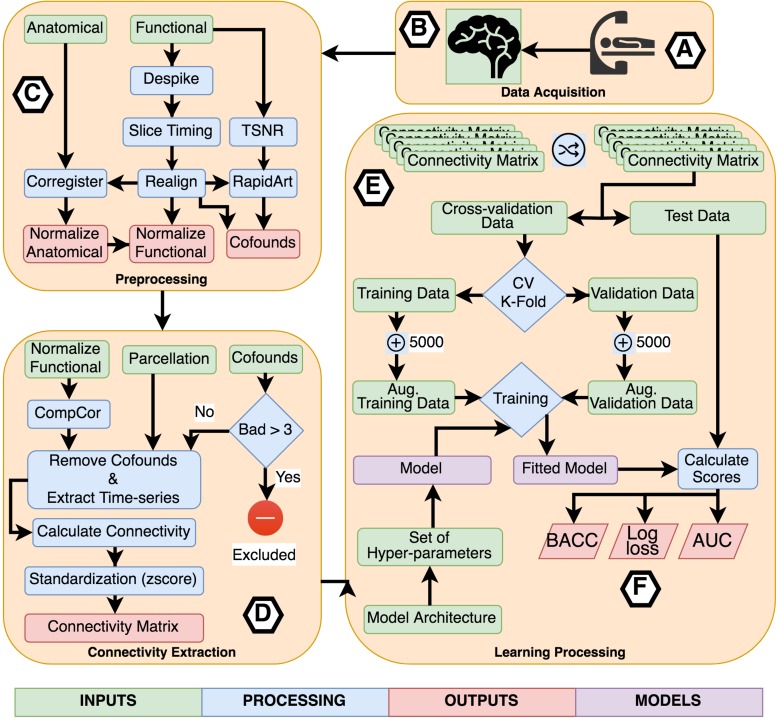
Flowchart describing the entire process of acquisition, preprocessing, processing, learning, and evaluation. **(A)** Participants were scanned in a resting-state protocol and **(B)** functional and structural images were collected. **(C)** Each subject’s images were preprocessed separately applying standard procedures like time-slicing, realigning, coregistration, artifacts detection, and normalization. **(D)** A connectivity matrix was created for each subject combining a normalized functional image, a set of confounds, a parcelation, and a connectivity measure. **(E)** The learning processing starts shuffling the list of subject’s connectivity matrix, we preserved the shuffling index to be replicated with the other inputs set combining the different parcelations and connectivity measures. The next step was to select the independent fold used to compare the different fit models. The remainder data was used in the cross-validation process. All models were trained using a k-fold cross-validation scheme with *k* = 2 to CNN based models and *k* = 5 for TOPT models. Inside the cross-validation process, from each testing and validation’s dataset, five thousand synthetic connectivity matrices were created. These synthetic data were used to train and validate the model, which is a combination of the model architecture and a specific set of hyper-parameters, including regularization. For the last, **(F)** the fitted model was evaluated using the independent fold and three scores: Balanced Accuracy (BACC), Log loss, and the Area Under the receiver-operating characteristics curve. These scores were used to compare the performance of architectures, parcelations, and connectivity measures.

For the experiments using TPOT, we applied a five stratified k-fold cross-validation process. Also, the genetic algorithm was configured to retain 50 individuals in every generation while running it for ten generations. Three scores were used as metrics to be optimized by TPOT: balanced accuracy, the area under the receiver operating curve (AUC), and log loss. Combining these three metrics with the eight connectivity matrices, we executed 24 classification experiments using TPOT. Figure 2 describes the entire process of acquisition, preprocessing, processing, learning, and evaluation.

## Results

In order to evaluate and compare the experiments, we used three scores: balanced accuracy; the area under the receiver operating curve (AUC); and cross-entropy loss (log loss). Balanced accuracy is defined as the average of *recall* obtained on each class, which in turn is the proportion of actual *positives* that are predicted as *positives*. Imbalance groups do not affect this accuracy score. The AUC of a classifier is equivalent to the probability that the classifier will rank a randomly chosen positive instance higher than a randomly chosen negative instance (Fawcett, 2006). For both balanced accuracy and AUC, the values can range between 0 and 1; values close to 1 indicate better classifiers, and the scores are calculated using the predicted classes. On the other hand, log loss can range from 0 to +∞, where values close to 0 are better scores. It is calculated using the probability of an instance to belong to a target class. It is defined as the negative log-likelihood of the true labels given a probabilistic classifier’s predictions. The log loss can be interpreted as a measure of *certainty*, where a classifier that predicts a wrong class with a high probability is punished (Bishop, 2006).

### Correlation vs. Dynamic Time Warping

**FIGURE 3:**
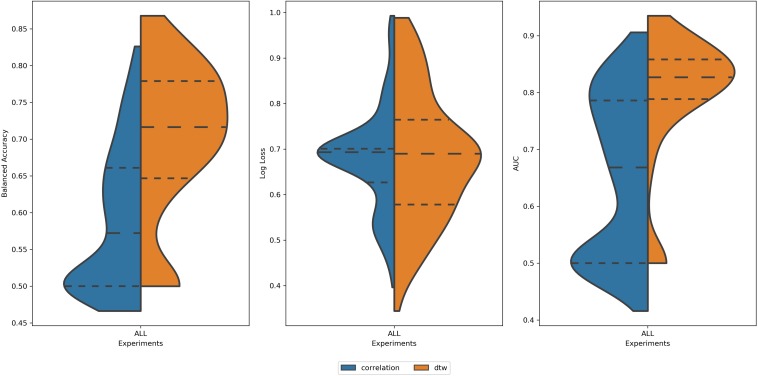
Distribution of values of **(left)** Balanced Accuracy, **(center)** Log Loss, and **(right)** AUC for all 2616 experiments using correlation (blue area) and DTW (orange area). All tails were truncated in maximum and minimum values while the dash lines delimit the quartiles. In the case of Balanced Accuracy and AUC, the distributions for Correlation and DTW differ with *p*-value < 0.05.

Analyzing all 2616 experiments, the first observed result was that DTW outperformed correlation in balanced accuracy and AUC (Figure 3). This result was found for the average score, as well for the best scores of each group. This result is in accordance with (Meszlényi et al., 2016). For the log loss, the difference between experiments using DTW and Correlation was not significant (Table 1). Thus, all the subsequent results will only summarize the experiments using DTW.

**TABLE 1:** Mean values of balanced accuracy, log loss, and AUC for experiments using correlation and DTW as a measure of connectivity.

	**Balanced accuracy**	**Log loss**	**AUC**
Correlation	0.62 ± 0.08	0.61 ± 0.10	0.73 ± 0.12
DTW	0.69 ± 0.09	0.60 ± 0.13	0.80 ± 0.9
t-index (*p*-value)	−1.89 (3.19e−66)	−0.95 (3.39e−01)	−16.3 (1.01e−51)

### Parcelation Size vs. Performance

**FIGURE 4:**
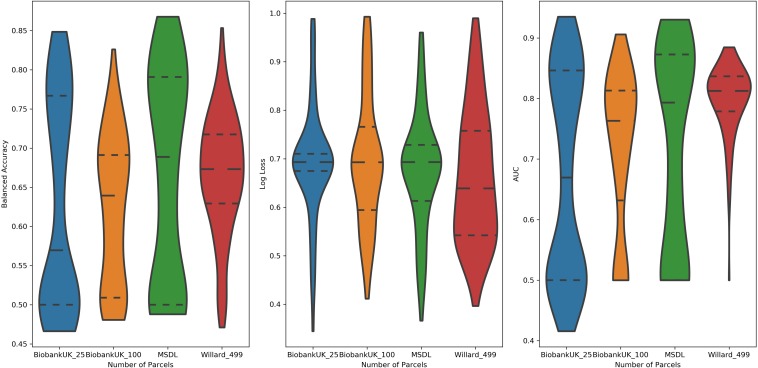
Distribution of values of **(left)** Balanced Accuracy, **(center)** Log Loss, and **(right)** AUC grouped by parcelation size: Biobank_UK_25 (blue), Biobank_UK_100 (Orange), MSDL (green), and Willard (red). All tails were truncated in maximum and minimum values while the dash lines delimit the quartiles.

Examining the performance of the brain parcelations, we observed that the number of parcels had no significant relation with the values of accuracy, AUC, and log loss (Figure 4). However, the parcelation seems to affect the performance of classifier separately. We can observe that both Ann4Brains architectures decrease (slightly) the performance with bigger parcelations. Meanwhile, the architecture proposed by Meszlényi et al. (2017) improves its performance with the increment of parcels. TPOT seems to be stable in relation to the parcelation size. Once TPOT finds the best pipeline in a set of different types of classifiers and/or dimensionality reduction techniques, it can adapt to the parcelation size.

### Best Classifier for Chronic Pain

**FIGURE 5:**
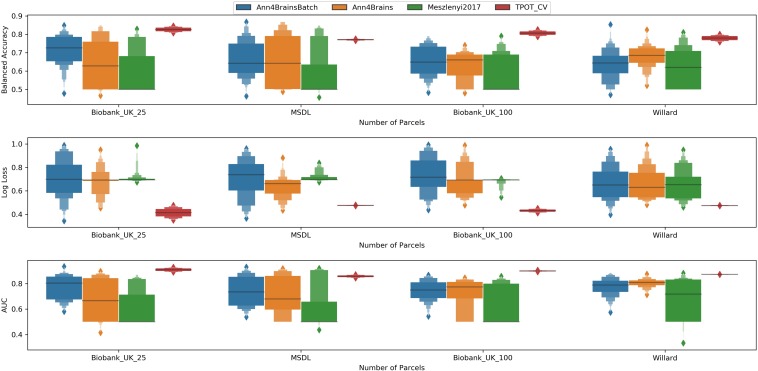
Enhanced box plot of values of Balanced Accuracy **(top)**, Log Loss **(middle)**, and AUC **(bottom)** grouped by parcelation size (horizontal axis) and classifier architecture: Ann4BrainsBatch (blue), Ann4Brains (orange), Meszlenyi2017 (green), and TPOT (red).

In Figure 5, we have a box plot with results of all experiments grouped by parcelation and architecture. TPOT clearly shows the best mean values for all metrics and parcelation. Since TPOT results are only for the best pipeline found by the genetic algorithm, higher mean (or lower in log loss case) was expected. Among the DL architectures, the Ann4Brains based architectures had better results compared with the architecture proposed by Meszlényi et al. (2017).

The best accuracy of classifying chronic pain was obtained using Ann4brains architecture and its variation with batch normalization (see Supplementary Figure 2). These classifiers exhibited accuracy values of 0.868. In both cases, the MSDL parcelation was used. Ann4brainsBatch also presented the best results for AUC and log loss, with values of 0.935 and 0.344, respectively. In that case, the BiobankUK_25 parcelation was used. In Table 2, we summarized the best results obtained by each classifier and parcelation. All these results were obtained using the DTW as a connectivity measure. The experiments using correlation had the best accuracy of 0.826 and the best AUC of 0.905 using TPOT and BiobankUK_100, which reinforce the results found in see section “Correlation vs. Dynamic Time Warping.”

**TABLE 2:** Best values of balanced accuracy, log loss, and AUC for each combination of classifier and parcelation using DTW.

	**Biobank_UK_25**	**MSDL**	**Biobank_UK_100**	**Willard**
Ann4Brains	BACC = 0.849	BACC = 0.868	BACC = 0.740	BACC = 0.825
	LLOSS = 0.453	LLOSS = 0.435	LLOSS = 0.479	LLOSS = 0.482
	AUC = 0.897	AUC = 0.918	AUC = 0.846	AUC = 0.875
Ann4BrainsBatch	BACC = 0.849	BACC = 0.868	BACC = 0.779	BACC = 0.853
	LLOSS = 0.345	LLOSS = 0.366	LLOSS = 0.438	LLOSS = 0.396
	AUC = 0.935	AUC = 0.930	AUC = 0.868	AUC = 0.885
Meszlenyi2017	BACC = 0.805	BACC = 0.849	BACC = 0.678	BACC = 0.810
	LLOSS = 0.676	LLOSS = 0.675	LLOSS = 0.546	LLOSS = 0.462
	AUC = 0.853	AUC = 0.916	AUC = 0.760	AUC = 0.882
TPOT	BACC = 0.846	BACC = 0.776	BACC = 0.826	BACC = 0.801
	LLOSS = 0.351	LLOSS = 0.472	LLOSS = 0.412	LLOSS = 0.471
	AUC = 0.929	AUC = 0.872	AUC = 0.906	AUC = 0.874

A limitation of this study is that the chronic pain group presented only female participants. To exclude the possibility that our models were learning to classify the groups according to participants’ sex rather than to the presence or not of chronic pain, all models were assessed by using sex as the target variable.

Supposing that the models were classifying the groups based on participants’ sex. It would be expected that the models had a better performance if evaluate using the sex label as the classification target. Evaluating this possibility using the independent fold, the mean result for balanced accuracy, log loss, and AUC were 0.67, 3.76, and 0.73, respectively. More specifically, the best model using DTW, MSDL, and Ann4BrainsBatch had, respectively, a balanced accuracy, a log loss, and AUC of 0.70, 1.69, and 0.78 using the sex label. Comparing with the chronic pain prediction, the higher values of log loss is an indication that if the models were learning the sex difference it would be predicting the wrong classes with high probabilities. In other words, the models are uncertain about the sex label. These results gave us the confidence that our models were not affected by the sex group limitation.

## Discussion

In this paper, we presented a classifier for chronic pain conditions using resting-state fMRI and one convolutional neural network architecture. This classifier, with a DL approach, outperformed traditional machine learning techniques. Also, in the search to find the best classifier, we performed a set of experiments to understand how parcelation and connectivity measures affect the classification process. These experiments revealed that while the parcelation does not affect significatively the classification, the use of DTW significantly increase the classifier performance.

The findings of this study clearly show that functional brain images in association with DL can be used to differentiate chronic pain patients from pain-free controls. The best model using the Ann4brain architecture and MSDL parcelation had a balanced accuracy of 86.8% while the AUC was equal to 0.918. In the literature, other attempts to classify chronic pain using brain images have been made. Chronic low back pain (Baliki et al., 2011; Callan et al., 2014; Ung et al., 2014), and fibromyalgia (Sundermann et al., 2014; Robinson et al., 2015; López-Solà et al., 2017) are the most common syndromes that were studied, while temporomandibular disorder (Harper et al., 2016) and knee osteoarthritis (Baliki et al., 2011) are also present in previous studies.

A tool for classifying individuals with chronic back pain (CBP) was proposed by Callan and collaborators (Callan et al., 2014). They used voxel-level differences from 13 CBP patients and 13 pain-free controls during periods of both resting and electrical stimulation. By using Sparse Logistic Regression (SLR), they reported an accuracy of 92.3%. A similar accuracy percentage (93%) has been achieved by using a combination of three classifiers based on SVM and LR in 37 FM patients and 35 pain-free controls when painful stimuli were applied (López-Solà et al., 2017). Moreover, an SVM classifier was able to accurately classify 10 patients with myofascial-type temporomandibular disorders and 10 matched pain-free controls during painful pressure stimulation (Harper et al., 2016). Compared to previous studies, the present work focused on the comparison of different methods to classify participants into chronic pain patients and healthy controls based exclusively on functional resting-state data. Furthermore, we were able to analyze the results from several classifiers for two different types of chronic pain conditions: FM and chronic back pain. Sundermann et al. (2014) used only resting-state images to identify potential functional connectivity among patients with FM, rheumatoid arthritis (RA), and pain-free controls. The author tried different types of classifiers based on SVM, decision tree, naive Bayes classifiers, etc. Comparing FM with RA and using a k-NN based classifier the best accuracy of 79% was reached. Meanwhile, the best accuracy comparing FM and pain-free controls only archived 73.5% with an SVM based classifier. Comparing with our work, the main difference is the absence of DL algorithms in the previous study. But, even so, if compared with our results using TPOT, our work outperforms the results found by Sundermann et al. (2014). Similarly, studies that used only structural images to classify chronic pain had a lower performance (Baliki et al., 2011; Ung et al., 2014; Robinson et al., 2015). The best result obtained by Baliki et al. (2011) was a balanced accuracy of 81.25% in a multi-class problem involving patients with CBP, complex regional pain syndrome (CPRS), Osteoarthritis (OA), and pain-free controls.

Another important finding of this study was that DTW outperforms Correlation when applied to measure the connectivity among brain areas. This result matches the results found by Meszlényi et al. (2016), where the authors demonstrated that DTW emphasizes group differences resulting in a better classification. Meszlényi et al. (2016) used a unique brain parcelation with 90 ROIs (Shirer et al., 2012), and two classifiers based on SVM and the LASSO. In this scenario, DTW and correlation were evaluated in two tasks: classify the correct gender and identify ADHD patients. Our analysis expands the work proposed by Meszlényi et al. (2016), showing that DTW outperforms Correlation in different scenarios composed of four types of parcelations. Moreover, with the aid of TPOT toolkit, we tested both connectivity measures for a variety of classifiers, including linear and non-linear approaches. The tests demonstrated that DTW had better results than Correlation.

The interpretability of neural networks is a challenge due to its “black-box” characteristics. Some efforts like the Lime tool tries to make an approximation of the neural network behavior and provide some interpretation about how the neural networks do its prediction. Unfortunately, tools like that are adapted to networks that use a typical convolutional filter with a 3 × 3 or 5 × 5 matrix convolving. These typical convolutional networks are specialized to identify edges, contrast, border, etc. In our case, our image is, in reality, a connectivity matrix and the neighborhood of a cell does not represent any relationship, requiring specialized convolutional filters. Because of that, we cannot make use of tools like Lime to interpret the prediction. In contrast, we can apply Lime to interpret the results from TPOT.

**FIGURE 6:**
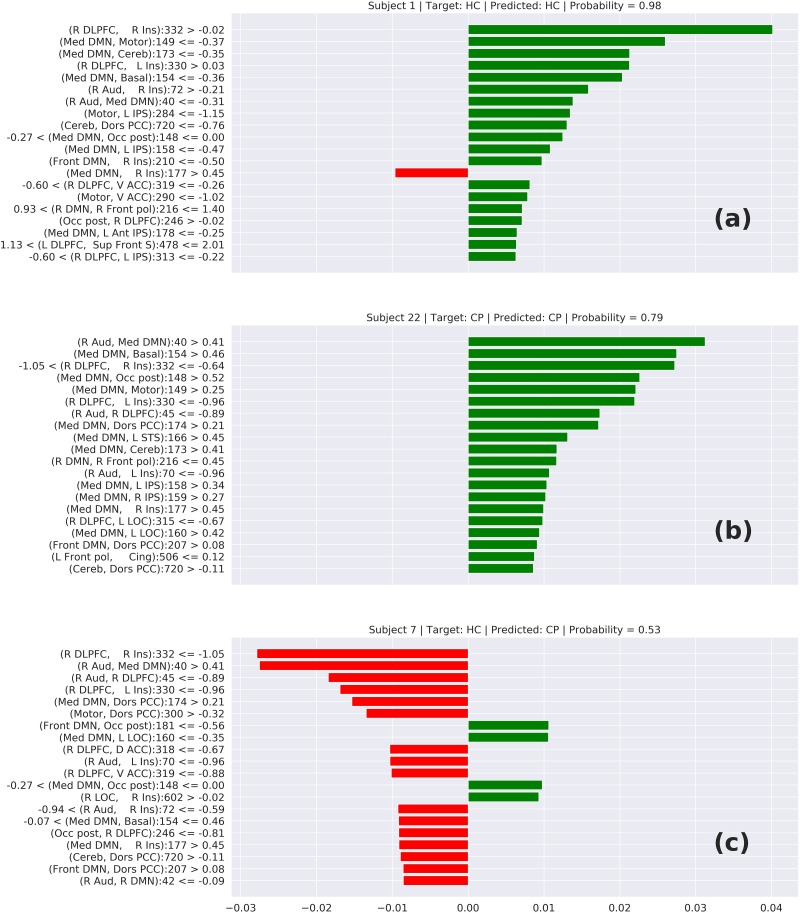
Local interpretable model-agnostic explanations (LIME) for the best TOPT model listing the 20 features that most contribute to the prediction of **(a)** a correctly predicted HC, **(b)** a correctly predicted CP, and **(c)** a wrongly predicted HC. The bars represent how much each feature (axis *Y*) added to the prediction in terms of probability. Green bars indicate that the feature adds positively, in direction to the target class, while the red bars indicate that the features are in resistance to the target class.

In Figure 6, we can see three cases of prediction: (6-a) HC correctly predicted, (6-b) CP correctly predicted, and (6-c) a wrong prediction. The features on top represent the elements that most contributed to this prediction. In the cases (6-a) and (6-b), we can notice that these top features have a connection involving the DMN or Insula, which suggests that the model is classifying the chronic pain mainly based on the connectivity of these two areas. This behavior is supported by other studies that relate these two areas with chronic pain syndromes (Baliki et al., 2008; Cauda et al., 2009). Analyzing the wrong prediction (6-c) we also can notice the presence of the DMN and Insula on the top features. But, in that case, the model predicted this HC subject as a CP subject with a lower probability of approximately 0.53. This number close to 0.5 means that the model was not confident about this prediction. Based on this interpretation, we can conclude that the model is making the predictions based on areas of the brain related to chronic pain.

A clear innovation of our work was the classification of chronic pain syndromes using neuroimaging and DL techniques. There are no records in the literature of any successful classification of chronic pain syndromes using a similar approach. This approach was already applied for other syndromes like attention-deficit and hyperactivity disorder, mild cognitive impairment, schizophrenia, Alzheimer’s disease (AD), and others (Du et al., 2018). Also, some previous studies compared DL techniques with a kernel-based model like SVM classifiers (Vieira et al., 2017). The majority of the studies show that when the overfitting problem is controlled, DL techniques outperform SVM classifiers. This behavior was also observed in our experiments, where the DL classifier (Ann4BrainsBatch) had the highest accuracy and AUC. Due to the small number of samples, the major challenge was to control the overfitting. To facilitate the process, L2 regularization (0.01) and a dropout rate of 0.5 were used between the hidden layers. Despite that, based on our results, we can conclude that DL techniques can provide good results in the task of classifying chronic pain syndromes.

Two possible limitations of the present study were (1) the different number of participants in the groups (chronic pain patients vs. pain-free controls), and (2) the fact that only women composed the group of chronic pain patients. To address the first limitation, a metric was used that is not affected by the differences in the number of subjects that make up the groups. In the case of the second limitation, the same analyses were performed but using the sex variable as the target to classify the participants. The levels of balanced accuracy, log loss, and AUC obtained with this variable as classifier were lower than those obtained with the variable “presence of chronic pain,” which reinforces the idea that our method worked better to classify according to chronic pain instead of sex.

Nevertheless, it would be necessary to evaluate how these techniques perform in a multi-class problem differentiating not only between pain-free and chronic pain but identifying different chronic pain syndromes. Moreover, investigating the possibility to predict a syndrome, a longitudinal study of a population with the risk of developing chronic pain could permit the application of an emerging and promising technique such as personalized machine learning (Rudovic et al., 2019).

## Data Availability Statement

The datasets for this article are not publicly available because other studies have been conducted with the same data and they were not published yet. Requests to access the datasets should be directed to PM, pedro.montoya@uib.es.

## Ethics Statement

The studies involving human participants were reviewed and approved by the Research Ethics Committee of the Balearic Islands. The patients/participants provided their written informed consent to participate in this study.

## Author Contributions

All authors listed have made a substantial, direct and intellectual contribution to the work, and approved it for publication.

## Conflict of Interest

The authors declare that the research was conducted in the absence of any commercial or financial relationships that could be construed as a potential conflict of interest.

## Footnotes

1 https://afni.nimh.nih.gov/pub/dist/doc/program_help/3dDespike.html

2 http://www.nitrc.org/projects/rapidart/

3 https://www.fmrib.ox.ac.uk/ukbiobank/

4 http://epistasislab.github.io/tpot/
