# Supplementary material for: Using Deep Learning and Resting-State fMRI to Classify Chronic Pain Conditions
Source: Front Neurosci. 2019 Dec 17;13:1313. doi: 10.3389/fnins.2019.01313 (PMC6929667; doi:10.3389/fnins.2019.01313)
Supplement: Supplementary file 1 [file Table_1.DOCX]

***Supplementary Material***

#
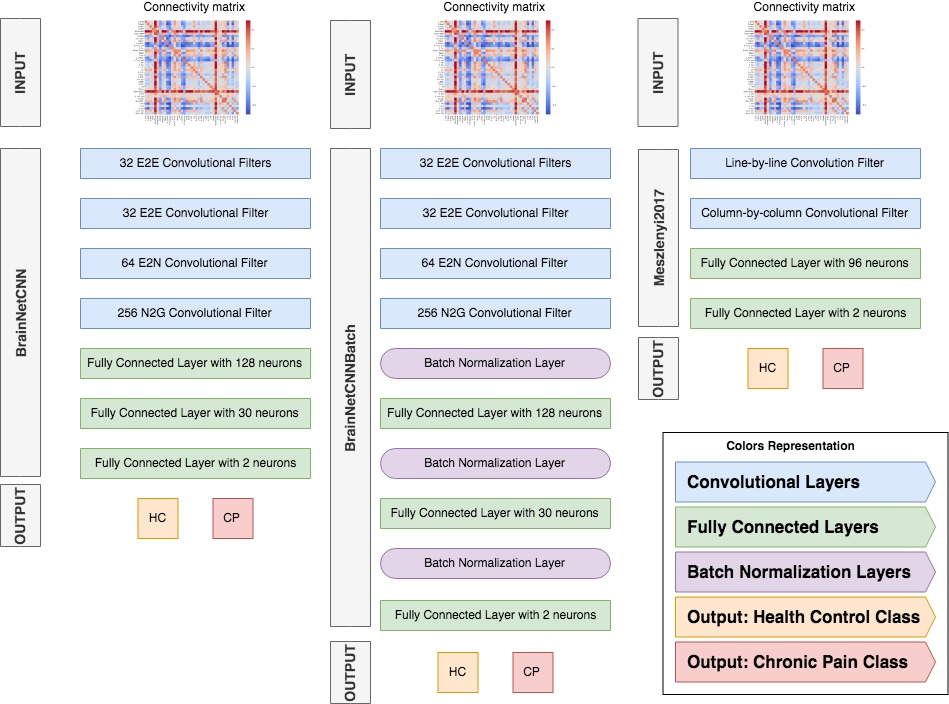
Neural Network Architectures and best classifier architecture.

**Supplementary Figure 1.** All neural network architectures used in our study: The BrainNet Convolution Neural Network (left), the BrainNet variation using batch normalization layers (middle), and the architecture purposed by (R. J. Meszlényi, Buza, and Vidnyánszky 2017) (right). The network data flows from top (input) to bottom (output).

**
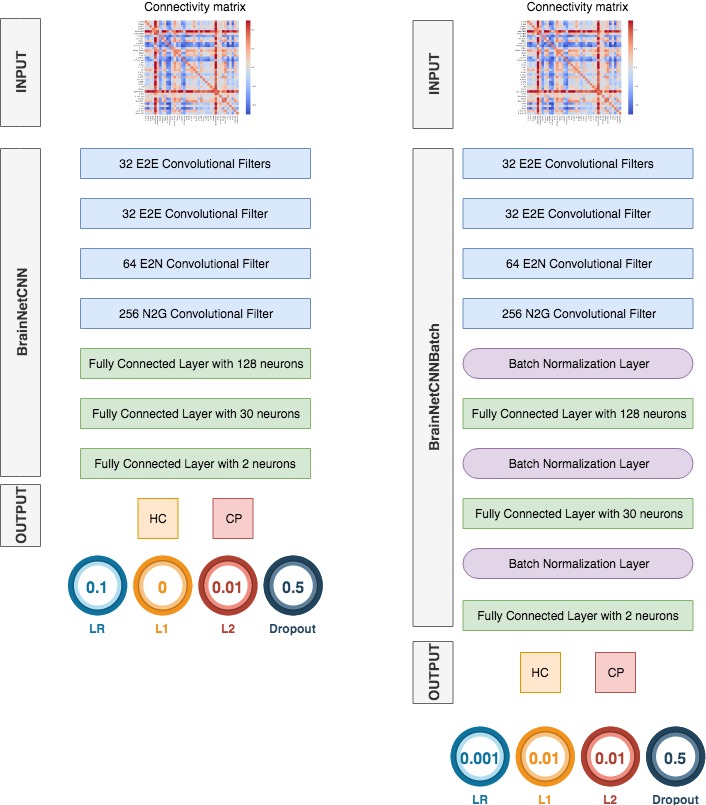
**

**Supplementary Figure 2.** The architectures and hyper-parameters of the two best classifiers. Both, BrainNetCNN (left) and BrainNetCNN with Batch Normalization layers (right) had the best value of balanced accuracy (0.868) using the MSDL Atlas and DTW as connectivity measure.
